# Effect of the implementation of infection prevention measures by an infection prevention link physician in trauma/orthopaedics surgery on hygiene-relevant processes and nosocomial infections

**DOI:** 10.1016/j.infpip.2024.100415

**Published:** 2024-10-26

**Authors:** Meike M. Neuwirth, Benedikt Marche, Jerome Defosse, Frauke Mattner, Robin Otchwemah

**Affiliations:** a Institute of Hygiene, Cologne Merheim Medical Center University Hospital Witten/Herdecke, Cologne, Germany; b Department of Hygiene and Environmental Medicine, Witten/Herdecke University, Cologne, Germany; c Division of Trauma and Orthopaedic Surgery, Cologne Merheim Medical Centre, University Hospital Witten/Herdecke, Cologne, Germany; d Department of Anesthesiology and Intensive Care Medicine, Cologne Merheim Medical Centre, University Hospital Witten/Herdecke, Cologne, Germany

**Keywords:** Nosocomial infections, Surgical site infection, Implementation, Infection prevention measures, Hand hygiene

## Abstract

**Background:**

The German Infection Protection Act and KRINKO recommend nominating one authorized medical specialist in every medical department as an infection prevention link physician (PLP). Detailed evidence on the contribution of PLPs to reducing infection rates is not available in Germany.

**Aim:**

The "HygArzt"-study investigated whether, and to what extent, a PLP in orthopaedics/trauma surgery is able to improve hand hygiene adherence (HHA), process steps of dressing change, nosocomial infection (NI) and surgical site infection (SSI) rates by implementing an infection prevention bundle (IPB).

**Methods:**

In consideration of a literature review on infection prevention measures in orthopaedics/trauma surgery and existing departmental hygiene standards, supported by the responsible infection control specialist, an IPB was developed by an interdisciplinary team and implemented by a PLP. The effects of IPB on NI, SSI, and HHA were determined in a pre-post study design on three trauma surgery/orthopaedic wards of a university hospital.

**Findings:**

In pre-post comparison HHA was significantly increased, and NI rates were reduced significantly. The greatest increase in adherence occurred in the pre-indications "Before touching a patient" (pre: 37.3%; post: 73.0%), "Before clean/aseptic procedure" (pre: 34.2%; post: 75.5%) and "Before surgery" (pre: 9.7%; post: 57.0%). The analysis of NI and SSI rates (NI: *p*=0.03; SSI: *p*=0.01; relative risk (RR) of 0.53 in each case) revealed rate reductions.

**Conclusion:**

The implementation of an IPB by a PLP led to an optimisation of processes and to a reduction of SSIs and NIs. PLPs seem to have the potential for targeted, group-specific implementation of complex IPBs.

## Background

Nosocomial infections (NI), particularly surgical site infections (SSI), may significantly impair patients' quality of life, mobility, and even result in death [[1], [2], [3], [4]]. They also increase the burden on healthcare systems by prolonging hospital stays and requiring further surgical interventions and leading to increased direct and opportunity costs. In Germany, direct costs related to SSI were recently estimated at approximately two billion euros per year [5].

Despite the existence of guidelines on infection prevention measures (IPM), such as those of the World Health Organisation (WHO), the Centers for Disease control and Prevention (CDC) or the Commission on Hospital Hygiene and Infection Prevention (KRINKO), and evidence-based proof of the effective reduction of SSIs through IPM, the number of SSIs has not decreased in recent decades [6]. One possible reason, cited for this lack of reduction, is that the required IPMs were often not consistently applied in everyday clinical practice [[6], [7], [8], [9]].

Implementing IPMs in clinical practice is a significant challenge [[10], [11], [12]]. Crucial factors for success comprise support from management staff, user confidence in the initiators of the new measures, the appropriate selection and tailoring of the measures to the specific local conditions and exposed pioneers during the implementation period of the measures from the ranks of the users (champions) [11,12].

As per the German Infection Protection Act and KRINKO recommendations, every medical department in Germany with a high risk of NI must employ an infection prevention link physician since 2011. She or he usually works in a leadership position within the medical department, acts as a mediator to the infection prevention team and acquires the necessary skills and knowledge through a 40-hour course. His/her duties include liaising with the Infection Prevention Department in the monitoring, transmission, detection and management of outbreaks (Ordinance on Hygiene and Infection Prevention in Medical Facilities (§ 5 Abs 1 und 2 *HygMed*)). PLPs might serve as institutionalized champions and possess information of department-specific processes and conditions, which might facilitate successful implementation of new IPMs.

The study hypothesises that infection prevention link physicians can successfully implement new IPMs, which may lead to a reduction in infection rates in an orthopaedic/trauma surgery department [13,14].

Although the effectiveness of implementing IPM in hospital departments by infection control nurses and infection prevention link nurses has been described [15], the role of infection prevention link physicians has not been systematically studied to date.

This study is part of the multicentre prospective cohort study 'HygArzt' and presents data from the pilot hospital [16].

## Methods

### Study design

In the monocentric part of the intervention cohort study presented here, the baseline situation before the intervention (pre-phase: 1st March 2018–31st August 2018) is compared with the results after the intervention (post-phase: 1st January 2019–30th June 2019) in three orthopaedic/trauma surgery wards in a university hospital. For further information on the methodological approach, see Neuwirth *et al.*, 2021 [16].

### Study population

All orthopaedic/trauma surgery patients who were treated as inpatients on a normal ward in a German university hospital and underwent surgery during the study period were included (Table I). Patients with pre-existing infections were excluded from the study. For additional inclusion and exclusion criteria and outcome parameters see Neuwirth *et al.*, 2021 [16].

**Table I:** Description of the study population: the pre- and post-phase

	Pre-intervention			Post-intervention		
	Number of patients	Hospital stays	Surgerys	Number of patients	Hospital stays	Surgerys
Total sample	1.324	1.413	1.676	1.319	1.401	1.772
Included^a^^,^^b^	1.211	1.413	1.430	1.269	1.401	1.583
Male	685	781	845	716	599	903
Female	526	576	585	553	802	680
Average age in years	-	-	48.85 (*SD =* +- 21.11; *MD* = 51)	-	-	50.99 (*SD =* +- 20,66; *MD* = 54)
Average ASA	-	-	2.09 (*SD* = +- 0,67; *MD* = 2)	-	-	2.016 (*SD =* +- 0,73; *MD* = 2)

SD = Standard deviation, MD = Median.

a A patient may have several operations and several stays.

b A single patient in the pre-intervention phase was counted twice (once for "acquired infection" and once for "acquired infections"), as this patient had an acquired SSI and acquired a further nosocomial infection during the stay.

### Process parameters

General hand hygiene of medical students, doctors and nurses was observed on the wards. General hand hygiene by doctors, theatre nurses and surgical assistants was observed in the operating theatre (OR). In addition, dressing changes, which were exclusively performed by doctors and medical students, were observed on the wards [16]. The observations were carried out by the infection prevention team.

### Intervention

During the pre-phase, SSIs were identified as the most common type of infection, hence, the intervention aimed onto SSI prevention. An IPB was developed in interdisciplinary and interprofessional collaboration (representatives from nursing, orthopaedics/trauma surgery, hospital hygiene), with individual evidence-based measures selected from a systematic literature review [15]. During the development process, the IPB was expanded to include measures to optimise existing processes, relevant to infection prevention, as well as new measures. The implementation of the IPB included theoretical training in the form of lectures, practical training in small groups on dressing changes, consisting of step-by-step procedure trainings and regular feedback of process parameters (HHA and dressing change adherence) to doctors and nurses by the infection prevention link physicians. The PLP was supported by the hygiene department in the preparation of the training materials and conducting the training sessions and was already present in the orthopaedics/trauma surgical department prior to the study.

The newly introduced measures included universal MSSA/MRSA admission screening, pre-operative antiseptic washing and use of antibiotic nasal ointment and use of remanent skin antiseptics prior to wound closure. Pre-existing measures, which were planned to be optimised, included post-operative wound care (among other things by the mounting of alcohol based hand disinfectant dispensers on patient beds of the wards), preoperative antibiotic prophylaxis and general hand hygiene.

In detail, patients were screened for methicillin-resistant *S.aureus* (MRSA) and methicillin-sensitive *S.aureus* (MSSA) by nasal swab at the time of registration for surgery. For elective patients, results were usually available prior to surgery. In case of MRSA identification, patients were decolonised before further treatment or treated under special precautions (contact precautions, vancomycin for antibiotic prophylaxis before surgery, antiseptic body washing, nasal ointment etc.). If MSSA was found, the decolonisation concept of the trial was implemented as in negative tested patients.

The infection prevention bundle included antiseptic body washing with Octenidin®1 (0.1% octenidine) and the use of mupirocin nasal ointment (2% mupirocin). As screening results were not available for most emergency patients at the time of surgery, all patients were decolonised according to the following scheme: Elective patients were asked to decolonise with Octenidin® wash lotion prior to surgery. Emergency patients were decolonised with Octenidin® washcloths (0.08% octenidine) by the nursing staff prior to surgery. Both, elective and emergency patients were given a nasal ointment containing mupirocin.

### Data collection

Checklists for recording hand hygiene on the ward and in the OR were used according to the "Aktion saubere Hände" (ASH), a nationwide, German hand hygiene compliance monitoring and improvement program, and supplemented with gender and time data.

For the standardised recording of hand hygiene and the process parameters of dressing changes, a checklist was drawn up during the preparatory phase based on existing publications [[17], [18], [19]].

In the pre-phase, medical staff were informed about the adherence observations and asked to follow their daily routine and ignore the observers. No additional instructions were given on how to perform the medical activities correctly.

In the intervention phase, the aim of the study was communicated and, if requested, bedside corrections and explanations were given on the correct performance of dressing changes, the recognition of hand hygiene indications and the performance of the measures previously practiced in theoretical and practical training by the PLP.

Theoretical training on dressing changes, observation results from the pre-phase on hand hygiene and current infection rates, particularly post-operative wound infections, as well as new measures from the IPB were carried out on three dates during the doctors' early morning meeting by the PLP. Practical training on dressing changes was conducted on various dates during the intervention phase so that every doctor in the department had the opportunity to attend.

During the post-phase, medical staff were observed without any corrections or training. The staff received feedback on their adherence to hand hygiene and the individual process steps of the dressing change weekly through postings in the ward staff rooms and every two weeks via email. Adherence was calculated by dividing the number of activities performed by the total number of activities required. Incorrectly performed activities were classified as not performed. Adherence observations were conducted by five trained observers.

The following operations of high-risk injuries were defined as indications for the applications of closed incision negative pressure wound therapy (CiNPWT (VAC dressings)): Calcaneus fractures, tibia plateau/pilon fractures, trimalleolar ankle fractures, if in addition at least one patient risk factor (diabetes mellitus, ASA score > 2; operating time > 90 min (incision-suture time), revision surgery, obesity per magna (≥ BMI of 40 kg/m^2^), age > 65y.).Acetabular fracture operations, lower limb amputations, open fractures and revision arthroplasties were generally classified as CiNPWT indications, regardless of additional risk factors [20]. The number of correct VAC applications was compared to the total number of operations performed, with at least one of the mentioned criteria being fulfilled.

The performance of preoperative washing and application of nasal ointment was extracted from the nursing documentation exclusively and was collected only during the post phase.

The project team defined NI according to the KISS and CDC definitions [21,22]. The study documented postoperative wound infections, nosocomial urinary tract infections (UTIs), nosocomial thrombophlebitis (including vascular catheter insertion site infections), primary sepsis, and Clostridioides difficile-associated diarrhoea (CDAD) [16]. Additionally, the rates of surgical site infections (SSIs) regarding different anatomical locations were determined.

Pre/post comparisons were utilised to calculate the relative risks (RR) and 95% confidence intervals (95% CI) of infection rates. The significance of adherence to hand hygiene, dressing changes and introduction of new measures was tested using the Chi-square test of independence (χ^2^-test). The significance level was set at p <0.05. A measure of the effects was calculated as the phi value (φ).

## Results

**Figure 1:**
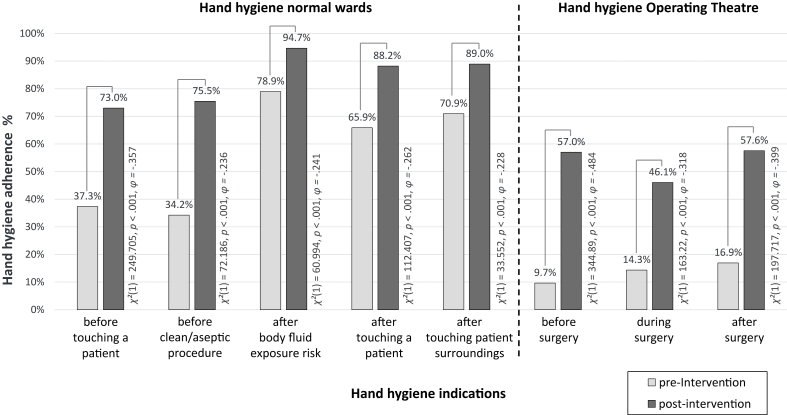
Adherence rates to hand hygiene in the operating theatre and on the ward in pre-post comparison (source: own figure).

Hand hygiene adherence rates on the wards showed a significant increase for all indications (pre-phase: 3,453 observations; post-phase: 3,686 observations) (see Figure 1).

Hand hygiene adherence was observed during 95 surgeries with 1,581 hand hygiene indications in the pre-phase and during 108 surgeries with 2,707 hand hygiene indications in the post-phase. HHA increased significantly for all indications compared with the pre-phase (Figure 1). Results regarding optimization the dressing change are published elsewhere [19].

Details of the new IPMs introduced during the intervention phase are presented in Table II. Universal nasal screening for MRSA/MSSA detected 3 MRSA and 118 MSSA positive patients. One thousand and seventy-six patients were tested negative for MRSA or MSSA (Table II).

**Table II:** Adherence rates of hygiene measures. The adherence rates of newly introduced measures (screening, antibiotic nasal ointment, antiseptic body wash) was only was only collected in the post-phase and the adherence rate to correct antibiotic prophylaxis are shown as existing measures in pre-post comparison

Adherence of new measures post-phase^a^						
Measure	Number of necessary applications^b^	Total adherence	Number of necessary applications elective patients	Adherence elective patients	Number of necessary applications emergency patients	Adherence emergency patients
Screening of MRSA and MSSA	1401	86.0% (N = 1206)	965	90.0% (N = 868)	436	77.5% (N = 338)
Antiseptic body wash	1401	62.2% (N = 872)	965	73.26% (N = 707)	436	37.8% (N = 165)
Antibiotic nasal ointment	1401	62.2% (N = 871)	965	71.6% (N = 691)	436	41.3% (N = 180)
Antiseptic body wash and antibiotic nasal ointment	1401	59.7% (N = 837)	965	70.7% (N = 682)	436	35.5% (N = 155)
Screening, antiseptic body wash and antibiotic nasal ointment	1401	56.0% (N = 784)	965	67.3% (N = 650)	436	30.7% (N = 134)
Surgeries with necessary applications of VAC therapy	84	5% (N = 4)	-	-	-	-

Adherence existing measures in a pre-post comparison							
Measure	Number of necessary applications pre-phase^c^	Total adherence pre-phase	Number of necessary applications post-phase	Total adherence post-phase^3^	X^2^	*p*	Effect size
Application of preoperative prophylaxis	562	96% (N = 540)	816	94% (N = 769)	2.382	0.123	*φ =* 0.041
Application of preoperative prophylaxis 0–60 min before cutting	562	73% (N = 394)	816	77% (N = 589)	2.016	0.365	*V* = 0.039

a Adherence to the newly introduced measures was only determined in the post-phase; the administration of preoperative antibiotic prophylaxis and the timing of its administration were determined both in the pre-phase and in the post-phase.

b Number of necessary treatments refers to the number of hospitalisations.

c Number of operations with indication for preoperative antibiotic prophylaxis.

A total of 61 nosocomial infections were detected in the pre-phase (44 SSIs, 7 UTIs, 5 pneumonias, 2 patients with thrombophlebitis, 2 CDAD and 1 case of primary sepsis) and 34 in the post-phase (26 SSIs, 4 UTIs, 3 pneumonias and 1 CDAD). Infection rates for both NI and SSI were significantly reduced (NI: p = 0.03; SSI: p = 0.01), each corresponding to a lower relative risk of 0.53 (Table III) [23].

**Table III:** Infection rates of body parts in pre/post comparison: The infection rates of individual body parts are compared pre/post and the relative risk was determined for each body part

	Pre-intervention			Post-intervention			Pre-post -comparison	
	Number of included Surgeries	Number NI	Infection rate NI %	Number of included Surgeries	Number NI	Infection rate NI %	RR [95% CI]	*p*
Surgeries total NI^a^	1430	58	4.1%	1583	34	2.2%	0.53 [0.35;0.79]	0.002∗∗
		Number SSI	Infection rate SSI %		Number SSI	Infection rate SSI %		

Surgeries total SSI^a^	1430	44	3.1%	1583	26	1.6%	0.54 [0.34;0.88]	0.01∗
Body part								
Pelvis^b^	10	0	0.0%	19	0	0.0%		
Hip	193	9	4.7%	168	1	0.6%	0.13 [0.02;1.00]	0.05∗
Thigh	71	2	2,8%	115	2	1.7%	0.62 [0.09;4.29]	0.63
Knee	430	6	1.4%	442	7	1.6%	1.14 [0.39;3.36]	0.81
Lower leg/foot	323	20	6.2%	411	13	3.2%	0.48 [0.24;0.94]	0.03∗
Shoulder	132	3	2.3%	131	1	0.8%	0.34 [0.04;3.19]	0.34
Collarbone^b^	39	0	0.0%	49	0	0.0%		
Upper arm	53	0	0.0%	75	1	1.3%	2.13 [0.09;51.34]	0.64
Elbow	37	1	2.7%	49	1	2.0%	0.76 [0.05;11.68]	0.84
Forearm	122	3	2.5%	86	0	0.0%	0.20 [0.01;3.86]	0.29
Spine^b^	15	0	0.0%	35	0	0.0%		
Thorax/rips^b^	0	0	0.0%	3	0	0.0%		

RR = relative risk, CI = Confidence interval, *p* = significance value, NI = nosocomial infection, SSI = surgical site infection.

∗= *p* ≤ 0.05 (significant), ∗∗ = *p* ≤ 0.01 (highly significant).

a The overall infection rates have already been published [23].

b RR could not be calculated as no infections occurred during the operations on this part of the body.

Infections with MSSA (pre N=19, post N=12), coagulase negative staphylococci (pre N=14, post N=8) and Enterobacterales (pre N=8, post N=4) were most frequently detected in wound infections in both the pre and post phases.

The highest infection rate was 6.2% after lower leg/foot surgery in pre-phase. In the post-phase, the risk of SSI on the lower leg/foot (RR=0.48 (CI 95% 0.24; 0.94), p=0.03) and hip (RR=0.13 (CI 95% 0.02; 1.0), p=0.05) was significantly reduced compared to the pre-phase (Table III).

Evaluation of SSI by sex in the pre-phase (♂=712; ♀=536; ♂= 35 SSI; ♀=9 SSI) showed a higher likelihood for men to develop SSI post-operatively (♂=35 SSI; ♀=9 SSI) (χ^2^(1) = 23.36, p<0.001, φ = 0.1). In the post-phase, no significant difference was identified between men and women in the likelihood of developing SSI (p = 0.83).

The overall rate of recorded nosocomial infections was 1.5% (CI 95% 0.8; 2.3) in elective patients and 4.6% (CI 95% 2.7; 6.5) in emergency patients in the post-phase, which corresponded to a lower risk (RR=0.32 (CI 95% 0.16; 0.62), p<0.001) for elective patients to develop NI.

No deaths occurred in either the pre- or post-phase.

## Discussion

The aim of this study was to investigate, whether an infection PLP in orthopaedic/trauma surgery can implement a tailored IPB and thus, reduce the rates of nosocomial infections in his/her department. The IPB was developed in interdisciplinary and interprofessional collaboration with the advice of the responsible hospital hygienist and implemented by the PLP. The implementation was monitored using various process parameters: HHA was significantly increased on the wards and in the OR and antiseptic washing and nasal decolonisation with mupirocin ointment reached an implementation level of 59.7%.

Perioperative AB prophylaxis was performed in accordance with the recommendations in 96% of cases in the pre-phase and the rate was maintained at the same level (94%). Adherence to nasal MRSA/MSSA screening was 86%. Adherence to ciNPWT was 5%. The introduction of IPB significantly reduced patients' risk of NI and SSI by 53% for NI and 53% for SSI compared to the pre-phase in our setting. The overall SSI rate for all patients in the pre-phase was 3.1% [23], which is comparable to the baseline rate in other studies (3.8%–5.8%) evaluating the effects of infection prevention interventions in orthopaedic/trauma units [[24], [25], [26]]. In addition, SSI rates and their modifiability differed according to the body region involved.

On normal wards, the HHA increased most significantly for the indication before clean/aseptic procedures during the post phase (pre: 34.2%; post 75.5%) and was thus higher than the national German reference value for normal surgical wards of 69% [24]. HHA also increased in the indication before touching a patient (pre: 37.3%; post 73.0%) and was higher than the German-wide reference value of 70% as well [27].

HHA observations in OR have rarely been published so far [28] and require an exact allocation of activities to the indication groups according to the WHO/ASH model. After the intervention, HHA increased most significantly in the operating theatre for the indication before surgery (pre: 9.7%; post 57.0%). In this study, there was no regular feedback of the HHA data in the OR and no practical training unlike the ward, which could explain the lower OR overall adherence.

The study's findings are in line with previous results, indicating that monitoring, feedback, and training can enhance HHA on hospital wards [27,[29], [30], [31]]. Additionally, the feedback provided to participants included HHA data from all three wards, which may have further increased motivation due to the resulting competitive environment [32].

During the intervention phase, bed dispensers for hand disinfectant were installed on patient beds on the wards. This increased the availability of disinfectants and indirectly reminded the medical staff to perform hand hygiene [[32], [33], [34]]. The increase in adherence may also be attributed to the targeted training on hand hygiene and the feedback provided by the PLP, which may have increased staff awareness of hand disinfection indications.

Another component of IPB was preoperative antiseptic body washing with Octenidin® (wash lotion or washcloths) and application of nasal ointment containing mupirocin (2%). The use of octenidine washcloths has led to controversial discussions in the literature [35].

Antiseptic body washing and antibiotic nasal ointment application were performed in 62.2% of all hospitalised patients (Table III), which represents a relatively high adherence rate compared to other published data (39%; 42.4%) [36,37]. Previous studies have shown the use of antiseptic washings and antibiotic nasal ointment can reduce SSI rates, although the specific adherence rates have not been explicitly published [8,9,24,38,39]. In this study, no increase in mupirocin resistance to staphylococci or other adverse effects due to mupirocin use was observed.

The comparison of infection rates between elective patients (1.5%) and emergency patients (4.6%) revealed a higher incidence of nosocomial infections in the latter group. This may partially be attributed to a lower adherence rate (35.5%) to antiseptic washing and application of antibiotic nasal ointment compared to elective patients, where adherence was 70.7%. The lack of preparation of emergency patients the day before surgery and the demanding care under emergency conditions may have been additional factors. For instance, higher wound contamination rates, such as in open fractures, are more likely to occur in an emergency context. Additionally, it is possible that antiseptic washings in the emergency department were not fully documented. To reduce infection rates among emergency patients or increase adherence to the measures, providing more frequent feedback, reminders, and monitoring might be helpful.

The results indicate that operations on the lower extremities (hip and lower leg) in orthopaedics and trauma surgery had a higher risk of surgical site infections (SSIs) compared to other body areas, which was published before [40]. The present work shows a significant reduction of SSIs and thus, effective infection prevention, particularly in the subgroup analysis of this high-risk body area.

Additionally, males had a higher risk of developing SSIs during the pre-phase. Male gender has already been identified as an SSI risk factor [41]. The measures implemented significantly reduced the risk of SSIs in men. Additionally, the number of other NIs recorded also decreased. However, due to the small number of cases, no statistically significant effects could be demonstrated for NIs other than SSIs.

A limitation of our study is that the observations on hand hygiene adherence on the ward and in the OR in both the pre- and post-phases were conducted exclusively during the early shift on weekdays. However, fewer dressing changes, ward rounds and operations are performed on weekends and during the late and night shifts, as fewer patients are admitted. The concentration of observations on the early shift may thus not have caused a significant bias.

The surveillance and adherence observations were conducted by the same individuals, which may have resulted in staff blindness. To avoid staff influence, a standardized surveillance protocol was implemented, utilizing the CDC/KRINKO hospital surveillance system's definition of nosocomial infections. Only events meeting these objective criteria were counted as infections.

The group of observed staff members remained relatively stable over the study period. So, the interpretation of adherence rate effect sizes should be viewed critically due to potential low internal validity. The presence of an observer may have led to experimenter effects, such as the Hawthorne effect, unintentionally improving HHA due to the observation situation in both study phases [42]. To minimize this effect, observers remained in the background and participants were asked to continue their usual routines. Additionally, it is possible that the effect may have weakened over the 15-month study period due to habituation. It is unlikely that this would have influenced the pre/post phase comparison, or if it did, it would have resulted in a tendency towards a lower HHA in the post phase.

The study did not allow a determination of the extent to which individual measures in the IPB were decisive in reducing NI rates. Furthermore, it did not compare conditions with and without the presence of an PLP, but rather assessed the potential of PLPs to implement an IPB.

Personnel requirements, such as skills, attitudes, support from management, commitment to change, and the initiator of the desired changes, are crucial for the successful implementation of an IPB and the resulting increase in adherence to hygiene measures, such as hand disinfection [36,43,44]. Local champions who propose and organize implementation are also important for introducing new measures [15]. The study indicates that appointing a surgeon as a PLP, primary stakeholder, and institutionalized local champion might be advantageous for implementing IPMs. The increase in adherence is likely due to the implementation of the infection prevention bundle, which was implemented and tailored to the target group by the PLP and led to a decrease in NI and SSI, especially in high-risk body areas. The bundle included essential components for a successful intervention, such as training, skill development, motivation, and system change [45].

## Conclusion

In summary, the introduction of IPB by PLPs is a promising strategy to target implementation, increase adherence rates to hand hygiene and process parameters, and reduce nosocomial infection rates.

## Author contributions

MN is the corresponding author, drafted this article and developed instruments to collect the study data, collected the data and analysed the study results.

BM conducted an extensive literature search and prepared a literature review on which the selection of the infection prevention intervention for the for the study bundle. He supported the implementation of the intervention measures.

JD provided the data from the anaesthesia database on the use of antibiotic prophylaxis and ASA score of the patients, participated in the interpretation of the data and contributed to the manuscript writing.

FM drafted and designed the study, critically revised this manuscript and is the scientific supervisor and director of the study.

RO prepared and designed the study, critically reviewed this manuscript and is scientific supervisor and leader of the study.

All authors read and approved the final manuscript.

## Ethics vote

No studies on humans or animals were conducted by the authors for this article. The ethical guidelines stated in each case apply to the studies listed. The ethics committee of Witten/Herdecke University was informed about the research project in the form of an ethics application and approved it (application no. 215/2017 dated 08.02.2018).

## Funding

This work was supported by the Federal Ministry of Health Germany under grant number (ZMVI1-2516FSB111). The Federal Ministry of Health Germany provided only personnel and material resources for the research project. The funding is a government grant.

## Conflict of interest statement

M. Neuwirth, B. Marche,. J. Defosse, F. Mattner and R. Otchwemah declare no conflict of interest.
